# The New Era of Stimuli-Responsive Hydrogels: Beyond Drug Delivery

**DOI:** 10.3390/pharmaceutics18080985

**Published:** 2026-08-10

**Authors:** Elena Giuliano, Donato Cosco

**Affiliations:** Department of Health Sciences and “AGreenFood” Research Center, University of Catanzaro “Magna Græcia”, Campus Universitario “S. Venuta”, I-88100 Catanzaro, Italy

## 1. Introduction

Over the past two decades, advances in polymer chemistry and biomaterial engineering have driven the development of hydrogels from simple hydrated polymeric networks into multifunctional formulations capable of responding dynamically to changes in their surrounding environment. Their ability to absorb large amounts of water or biological fluids while maintaining structural integrity, together with their excellent biocompatibility, tunable physicochemical properties and responsiveness to physiological or external stimuli, has extended their applications in the fields of drug delivery, tissue engineering, wound management and regenerative medicine [1,2].

Despite these remarkable advances, several challenges continue to limit the clinical applications of stimuli-sensitive hydrogels [3,4]. The optimization of mechanical strength without compromising injectability, the precise modulation of drug release kinetics, long-term stability, the manufacturing scalability and the reproducibility of hydrogel architecture remain important issues. Furthermore, the complexity of biological microenvironments often requires hydrogel systems capable of performing multiple functions simultaneously, including tissue adhesion, self-healing, antimicrobial activity and responsiveness to disease-specific stimuli. Addressing these challenges requires an integrative approach combining pharmaceutical technology, polymer chemistry, materials science and biomedical engineering.

The Special Issue “*Application of Stimuli-Sensitive Hydrogels for the Treatment of Human and Animal Diseases*” was conceived to provide an updated overview of recent advances in this rapidly evolving field. Although the contributions address different therapeutic applications, they collectively illustrate a common trend: the transition from conventional hydrogel formulations toward multifunctional and application-oriented smart biomaterials designed to improve therapeutic efficacy through responsive behavior.

## 2. Overview of Published Works

The future of stimuli-sensitive hydrogels is increasingly focused on multifunctionality, personalization and clinical translation [5]. In their review, Ünlü et al. describe this evolution in the context of microbial diseases, focusing on alginate- and chitosan-based smart hydrogels. These two polymers are presented as representative systems for examining how polymer composition, structural modification and physicochemical properties influence antimicrobial performance and therapeutic outcomes [5]. Particularly relevant is their discussion of dual- and multi-responsive hydrogels, which are increasingly considered promising platforms for overcoming antimicrobial resistance while minimizing systemic drug exposure [5].

Building on the concepts discussed in this review, Zhao et al. provide an example of how stimuli-responsive hydrogels can be applied to the treatment of microbial diseases [6]. The authors developed a multifunctional pH-responsive hydrogel based on carboxymethyl chitosan and oxidized hyaluronic acid for the treatment of vulvovaginal candidiasis (VVC). By incorporating *Lactobacillus plantarum* and *Lactobacillus rhamnosus*, both characterized by antifungal activity, the formulation utilized microbiota modulation as an alternative therapeutic strategy; the hydrogel significantly enhanced probiotic storage stability and enabled pH-triggered release in the acidic vaginal environment. It also showed good cytocompatibility and inhibited the proliferation of *Candida albicans*, reduced inflammation and promoted vaginal epithelial tissue regeneration [6]. These findings further demonstrate the versatility of stimuli-responsive hydrogels as therapeutic platforms capable of delivering a broad range of bioactive agents beyond conventional small-molecule drugs.

A different translational perspective is presented by Jacob and coworkers, who designed an injectable thermoreversible nanoemulsion-based hydrogel depot for prolonged lidocaine delivery [7]. This study exemplifies how the rational integration of multiple drug delivery systems can overcome the limitations of individual carriers [3,8]. By combining complementary technologies, the authors addressed the poor aqueous solubility of lidocaine and simultaneously achieved in situ depot formation for sustained local drug release [7]. The use of formulation components that are generally recognized as safe and approved for parenteral administration by both the U.S. Food and Drug Administration and the European Medicines Agency is of particular interest. This approach addressed one of the major obstacles to the clinical translation of multicomponent drug delivery systems, namely the need for extensive regulatory evaluation of newly introduced excipients [9].

Arpa and Biltekin Kaleli provide another example of the versatility of stimuli-responsive hydrogels by demonstrating how the same local anesthetic, lidocaine, can be adapted to a completely different clinical application [10]. They developed a sprayable thermosensitive formulation for topical wound management comprising chitosan and poloxamer 407, combining lidocaine for rapid analgesia with allantoin to promote tissue repair. The adoption of a sprayable thermosensitive formulation addresses practical limitations of conventional creams and ointments, particularly when treating painful or irregular wound surfaces, further illustrating how rational hydrogel design can expand the therapeutic potential of stimuli-responsive hydrogels [10,11].

Finally, the importance of rational hydrogel design is further emphasized in the study by Panainte and colleagues, who investigated how different crosslinking strategies influence the physicochemical and biological performance of hyaluronic acid-based matrices [12]. By comparing physically and covalently crosslinked systems, the authors demonstrate that formulation parameters critically determine bioadhesion, swelling behavior, enzymatic stability, drug release and cellular response, reinforcing the central role of formulation design in unlocking the versatility of hydrogel formulations [11,12].

## 3. Conclusions and Future Perspectives

The contributions collected in this Special Issue highlight the remarkable versatility of stimuli-responsive hydrogels across a broad spectrum of biomedical applications, ranging from antimicrobial therapy and local anesthesia to wound management and biomaterial design. Despite targeting different clinical challenges, all contributions share a common theme: the ability to tailor hydrogel performance through rational formulation and structural engineering. Together, these studies demonstrate that the convergence of materials science, pharmaceutical technology and biomedical engineering is driving the development of increasingly sophisticated hydrogel platforms capable of addressing complex therapeutic needs. The advances in this field are expected to accelerate the clinical translation of stimuli-responsive hydrogels, supporting the development of innovative therapeutic solutions for both human and veterinary medicine.
